# Coldness impedes tumor growth

**DOI:** 10.1093/lifemeta/loac022

**Published:** 2022-09-15

**Authors:** Shudi Luo, Xiaoming Jiang, Zhimin Lu

**Affiliations:** Zhejiang Provincial Key Laboratory of Pancreatic Disease, The First Affiliated Hospital, and Institute of Translational Medicine, Zhejiang University School of Medicine, Hangzhou, Zhejiang 310029, China; Cancer Center, Zhejiang University, Hangzhou, Zhejiang 310029, China; Zhejiang Provincial Key Laboratory of Pancreatic Disease, The First Affiliated Hospital, and Institute of Translational Medicine, Zhejiang University School of Medicine, Hangzhou, Zhejiang 310029, China; Cancer Center, Zhejiang University, Hangzhou, Zhejiang 310029, China; Zhejiang Provincial Key Laboratory of Pancreatic Disease, The First Affiliated Hospital, and Institute of Translational Medicine, Zhejiang University School of Medicine, Hangzhou, Zhejiang 310029, China; Cancer Center, Zhejiang University, Hangzhou, Zhejiang 310029, China

**The Warburg effect is critical for tumor growth. A new study showed that cold acclimatization activates brown adipose tissue (BAT), reduces blood glucose levels, and subsequently blunts aerobic glycolysis in cancer and tumor growth.**

Tumor cells preferentially use glucose as an energy resource even in the presence of ample oxygen, to generate ATP and metabolic intermediates for the production of amino acids, fatty acids and cholesterol, and nucleotides. This Warburg effect or aerobic glycolysis occurring in various types of solid tumors has been used for cancer diagnosis and monitoring tumor progression and response to therapies by positron emission tomography–computed tomography (PET–CT) imaging analysis, which detects the substantially enhanced uptake of ^18^F-fluorodeoxyglucose (^18^F-FDG) of tumor cells [1]. The Warburg effect is strongly increased by oncogenic signaling. Mutations or overexpression of receptor protein kinases (RTKs) promote aerobic glycolysis in tumor cells through distinct mechanisms, including conferring the moonlighting functions of glycolytic enzymes that increase the activities or expression of glucose transporter (GLUT) and glycolytic enzymes and coordinate the regulation of glycolysis and mitochondrial functions [1, 2]. For instance, RTK activation or hypoxia promotes nuclear translocation of pyruvate kinase isozyme M2, which forms a complex with transcription factors and promotes β-catenin-, c-Myc-, and hypoxia-inducible factor-1α-dependent expression of GLUT1 and multiple glycolytic enzymes [3–5]. RTK activation and mutation of K-Ras or B-Raf induces mitochondrial translocation of phosphoglycerate kinase 1 (PGK1), which acts as a protein kinase and phosphorylates and activates pyruvate dehydrogenase kinase 1, leading to suppression of mitochondrial pyruvate metabolism and enhancement of pyruvate utilization for lactate production [6]. The moonlighting protein kinase activity of PGK1 can autophosphorylate PGK1 at Y324 and autoactivate PGK1, and the deficiency or mutation of PTEN, which functions as a protein phosphatase to dephosphorylate PGK1 pY324, in tumor cells enhances the PGK1-promoted Warburg effect [7]. As a rate-limiting enzyme in glycolysis, the platelet isoform of phosphofructokinase 1 (PFK1) translocates to the plasma membrane, where it is phosphorylated at Y64 by epidermal growth factor receptor. Phosphorylated Y64 binds to the p85 SH2 domain, leading to the recruitment and activation of phosphoinositide 3-kinase (PI3K). The subsequently activated AKT phosphorylates phosphofructokinase 2 (PFK2) to produce fructose-2,6-bisphosphate for PFK1 activation and enhances GLUT1 membrane expression, resulting in promoted aerobic glycolysis [8]. Thus, the intrinsic signaling forms activate oncogenic proteins, or deficient tumor suppressors induce the Warburg effect. However, whether the Warburg effect can be extrinsically modulated by regulation of glucose levels in body systems in a diet-independent way is not clear.

A recent report showed that brown fat can compete with tumors for glucose uptake and that coldness tilts the balance toward enhanced utilization of glucose in brown fat with a corresponding decrease in aerobic glycolysis in tumor cells [9]. Cold exposure (4°C) of immunodeficient mice with xenografted tumors derived from subcutaneous implantation of colorectal cancer (CRC) cells, fibrosarcoma cells, breast cancer cells, melanoma cells, or pancreatic ductal adenocarcinoma cells strongly decreased tumor growth with prolonged mouse survival time compared to that of the mice under 22°C or 30°C conditions. Immunohistochemical analysis of tumor tissues showed that cold exposure reduced the expression of the proliferative marker Ki-67 and the hypoxic marker carbonic anhydrase 9, and the density of CD31^+ ^tumor microvessels. Cold acclimatization, which did not alter the core body temperatures of mice, also inhibited the growth of tumors in immunocompetent mice using the genetic spontaneous MMTV-PyMT breast cancer model and the spontaneous *Apc*^Min/+^ intestinal adenoma model, as well as a mouse model with implantation of mouse CRC cells into the liver. These findings indicated that coldness blunts tumor growth regardless of body temperature or the direct effect of low temperature by skin contact [9].

Cold acclimatization activates BAT to generate heat through nonshivering thermogenesis (NST), which is mediated by mitochondrial uncoupling protein 1 (UCP1), and glucose contributes to BAT thermogenesis [9]. As expected, mice with or without tumors all exhibited BAT activation under cold exposure, as reflected by deceased multilocular structures accompanied by correspondingly increased cytochrome C oxidase subunit 4 (COX4)^ + ^mitochondrial contents, CD31^ + ^ microvessel density, and UCP1 expression. Moreover, ^18^F-FDG PET–CT analyses of the mice showed that ^18^F-FDG primarily accumulated in the tumor tissues and was modestly detected in the BAT. Nevertheless, cold exposure strongly reduced glucose uptake in tumors with a notable increase in BAT glucose uptake, a decrease in the levels of fast blood glucose, increased insulin sensitivity, and quick glucose clearance. Surgical removal of BAT in mice restored ^18^F-FDG uptake in breast cancer and considerably abrogated tumor suppression by cold acclimatization with improved tumor hypoxia, angiogenesis, and tumor cell proliferation. Intriguingly, tumor-bearing mice administered 15% glucose in the drinking water showed abrogation of the cold exposure-induced tumor-inhibitory effect. These results revealed a critical role of cold-activated BAT in the reduction of blood glucose levels and the subsequent reduced glucose uptake by tumor cells and tumor growth. This finding was further supported by gene set enrichment and metabolomics analyses, which showed dramatic increases in glycolysis with elevated expression of GLUT4 and glycolysis-related genes in BAT under cold exposure. In contrast, glycolysis, the levels of GLUT1, GLUT4, and GLUT7, and the activities of PI3K, AKT, and mammalian target of rapamycin were significantly decreased in cold-exposed tumors.

Mitochondrial UCP1 is crucial for NST in adipose tissues. Ucp1 deletion reduced heat production and ablated cold acclimatization-suppressed tumor growth with restored glycolysis in tumors accompanied by nonsignificant glucose uptake in cold-exposed BAT. Nevertheless, how UCP1-mediated mitochondrial activity is linked to glucose uptake and glycolysis needs further investigation.

Notably, some mouse results were replicated in humans in a pilot study. Healthy volunteers (three males and three females) who were exposed at 16°C for 2–6 h per day for 14 consecutive days exhibited BAT activation in the bilateral areas of supraclavicular, cervical, and parasternal regions, as reflected by increased ^18^F-FDG uptake. Consistently, similar BAT activation was observed in an 18-year-old patient with Hodgkin’s lymphoma exposed to 22°C for 7 days and diminished by exposure to 28°C. Importantly, reduced glucose uptake in the tumor tissue under cold exposure was detected.

These findings uncover a mechanism underlying the regulation of blood glucose levels by cold exposure, which enhances the utilization of glucose by the BAT and reduces glucose availability to tumor cells. High glucose levels can increase PD-L1 expression in tumor cells for tumor immune evasion by HK2-mediated NFκB activation [10] and pancreatic insulin secretion, which in turn can activate PI3K-AKT in tumor cells to increase the Warburg effect [8]. The impact of BAT activation on the inhibition of tumor growth can be attributed to the integrated regulation of tumor cell metabolism, the immune response, and the activation of oncogenic signaling, and provides a rationale to intervene in cancer with cold acclimatization.
